# A novel colloidal gold immunochromatography assay strip for the diagnosis of schistosomiasis japonica in domestic animals

**DOI:** 10.1186/s40249-017-0297-z

**Published:** 2017-04-07

**Authors:** Rui Xu, Jintao Feng, Yang Hong, Chao Lv, Dengyun Zhao, Jiaojiao Lin, Ke Lu, Hao Li, Jinming Liu, Xiaodan Cao, Tao Wang, Jinli Zai, Zhaozhe Wang, Bingguang Jia, Qian Han, Chuangang Zhu

**Affiliations:** 1 Key Laboratory of Animal Parasitology, Ministry of Agriculture of China, Shanghai Veterinary Research Institute, Chinese Academy of Agricultural Sciences, No.518, Ziyue Road, Minhang District, Shanghai 200241 China; 2 Jiangsu Co-innovation Center for Prevention and Control of Important Animal Infectious Diseases and Zoonoses, Yangzhou, 225000 China

**Keywords:** Colloidal gold immunochromatography assay strip, *Schistosoma japonicum*, Recombinant streptococcal protein G, Immunodiagnosis

## Abstract

**Background:**

Schistosomiasis remains a major public health concern in China and an epidemiological survey has revealed that schistosome-infected bovines and goats are the main transmission sources for the disease. Therefore, development of a sensitive technique for the diagnosis of schistosomiasis in domestic animals is necessary.

**Method:**

A novel colloidal gold immunochromatography assay (GICA) strip was developed for detecting *Schistosoma japonicum* in domestic animals. The colloidal gold was conjugated with recombinant streptococcal protein G (rSPG). As the test and control lines, the schistosome soluble egg antigen and rSPG, respectively, were blotted on nitrocellulose membrane.

**Results:**

The lowest detectable serum dilution was 1∶640 for schistosome-infected buffaloes. The cross-reaction rate of GICA was 14.29% with *Paramphistomum* sp. in buffaloes, 16.67% with *Haemonchus* sp. in goats, and 33.33% with *Orientobilharzia* sp. in goats. These results were slightly lower and similar to those obtained through ELISA. Moreover, the strips for detecting *S. japonicum* in mice, rabbits, buffaloes, and goats showed high sensitivity (100.00%, 100.00%, 100.00%, and 100.00%, respectively) and specificity (100.00%, 100.00%, 94.23%, and 88.64%, respectively). And the sensitivity or specificity of the GICA strips did not present any significant differences after storage for 12 months at room temperature. When compared with ELISA, the GICA strips exhibited similar sensitivity and specificity in the diagnosis of schistosomiasis in mice, rabbits, buffaloes, and goats. Besides, only 5 μl of serum are required for the test and the detection can be completed within 5 min.

**Conclusion:**

This study is the first to develop a GICA strip using gold–rSPG conjugate for the diagnosing of schistosomiasis in domestic animals, and preliminary results showed that the developed strip may be suitable for large-scale screening of schistosomiasis in endemic areas.

**Electronic supplementary material:**

The online version of this article (doi:10.1186/s40249-017-0297-z) contains supplementary material, which is available to authorized users.

## Multilingual abstracts

Please see Additional file 1 for translations of the abstract into the six official working languages of the United Nations.

## Background

Schistosomiasis is a serious zoonotic parasitic disease, causing considerable problems in many tropical and developing nations [1]. The World Health Organization has estimated that 258 million people required preventive treatment for schistosomiasis and that schistosomiasis transmission has been reported in 78 countries [2]. Schistosomiasis is caused by *Schistosoma japonicum* and remains a major public health problem in China, significantly affecting economic and public health [3, 4]. Despite over 50 years of concerted campaigns for controlling schistosomiasis epidemics, the disease still poses a major public health challenge in China [5, 6]. The threat of schistosomiasis constantly exists because most of the areas in China in which it is endemic have been characterized by low-intensity infection that is independent of prevalence.

Currently, *S. japonicum* infections are usually detected by parasitological or immunological methods. The parasitological methods include stool egg examination and fecal miracidium hatching test, which are the gold standards for the diagnosis of schistosomiasis in domestic animals. However, the sensitivity of parasitological methods is compromised for subjects with low-intensity infections and in areas with low prevalence of infection [7]. With regard to immunological methods, ELISA is the most widely used technique [8]. However, some limitations, including the need for expensive equipment and reagents, appropriate laboratory facilities, and technical expertise, hinder its application in community surveys. Thus, both parasitological and traditional immunological (ELISA) methods are not conducive for the detection of *S. japonicum* infection on a large scale.

In contrast, the colloidal gold immunochromatography assay (GICA) is simple, rapid, sensitive, and specific, does not need any special equipment, and can be applied for large-scale screening in epidemic areas. In most of the serological detection methods, the schistosome soluble egg antigen (SEA) has been employed as the source of target antigen. In addition, staphylococcal protein A (SPA) conjugated with colloidal gold has been commonly used in recent times. Nevertheless, when compared with SPA, streptococcal protein G (SPG) has a higher affinity for IgG binding and a wider application [9].

Thus, in the present study, we developed recombinant SPG (rSPG) containing only the C3 domain and conjugated it with colloidal gold to obtain rSPG–gold. By using SEA and rSPG, we developed and evaluated the GICA strip for the detection of *S. japonicum*.

## Methods

### Serum samples

Serum samples were collected from 50 mice, 30 rabbits, and 18 buffaloes that were artificially infected with *S. japonicum* and from 20 mice and 20 rabbits that were healthy and without infection. This 18 buffaloes were artificially infected with *S. japonicum*. All buffaloes were sacrificed 6 weeks post-challenge by portal perfusion. Worms in the perfusate sediment were collected and counted, and the intestinal mesenteric vessels of each mouse were examined for residual worms. According to the number of worms, we had carried on the grouping to buffaloes. In addition, serum samples were collected from 73 goats and 80 buffaloes, which presented hatching *S. japonicum* miracidia in their stool, as well as from 44 goats and 52 buffaloes from schistosomiasis-non-endemic areas. Furthermore, serum samples were collected from 37 *Orientobilharzia*-positive goats in which the parasites were found in their portal venous system, 12 *Haemonchus contortus*-positive goats in which the parasites were found in their abomasa, and 14 *Paramphistomum*-positive buffaloes in which the parasites were found in their portal venous system.

### Cloning and expression of rSPG

**Fig. 1:**
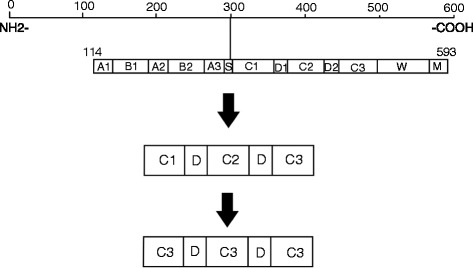
SPG transformation process

The C domain of protein G was determined from the GenBank sequences of SPG, and the rare codons of the sequences were replaced with *E. coli*-preferred codons. Subsequently, the C1, C2, C3, and D domains were found, and the C1 and C2 domains were replaced with the C3 domain (Fig. 1). The rSPG was obtained from our previous study [10].

### Western blot analysis

The rSPG in the gel was transferred onto the NC membrane, blocked using PBST (PBS with 0.05% (w/v) Tween–20) with 5% skim milk at room temperature for 2 h, and washed thrice with PBST for 10 min, and incubated for 2 h with HRP-conjugated goat anti-rabbit IgG (diluted with PBST at a ratio of 1∶2 500) at 37 °C. Then, after three washes, the membrane was visualized using an enhanced HRP-DAB kit (Tiangen Biotech, Beijing, China).

### Affinity constant of rSPG with the IgG from different animals

To determine the affinity constant (*K*_a_) of rSPG with the IgG from different animals, microtiter plates (Costar, Acton, MA, USA) coated with rSPG and SPG, respectively, were incubated overnight at 4 °C. Then, rSPG and SPG were diluted with carbonate bicarbonate buffer (pH 9.6) to 10, 5, 2.5, 1.25, 0.625, 0.313, 0.156, and 0.078 μg/ml, respectively. Three wells were coated with each dilution, respectively. The plates were blocked with 1% (w/v) gelatin/PBST for 2 h at 37 °C and then washed thrice with PBST for 10 min. Subsequently, the HRP-conjugated goat anti-mouse, bovine anti-mouse, mouse anti-rabbit, and rabbit anti-chicken IgGs were serially diluted with PBST to a ratio of 1∶500, 1∶1 000, 1∶2 000, and 1∶4 000, respectively, were added to the wells (100 μl/well) and the plates were incubated at 37 °C for 2 h. After three washes, 3,3′,5,5′-tetramethyl benzidine dihydrochloride was added to the plates (100 μl/well) and the reaction was stopped after 10 min using 2 M sulfuric acid (50 μl/well). The OD of the wells at 450 nm was determined using a microplate reader (Tecan, Mannedorf, Switzerland).

Using the OD measured at 450 nm as the ordinate and the logarithm of the antibody concentration as the abscissa, and based on the fitted curve and formula, *K*_a_ = [Ag/Ab]/([Ag][Ab]), the value of *K*_a_ was calculated and the average *K*_a_ values of rSPG and SPG were obtained.

### Preparation of colloidal gold–rSPG conjugate

The colloidal gold suspension was obtained from our previous study [10]. Then, approximately 1.2 ml of purified rSPG (1.0 mg/ml) was gently added into 100 ml of gold colloid solution (pH 6.0) under slow stirring. Then, the mixture was vigorously stirred for 30 min and 10 ml of 10% (w/v) poly (ethylene glycol) 20 000 solution were added to block the reaction of gold colloid, and the mixture was stirred again for 30 min. Subsequently, the mixture was centrifuged at 3 000 × *g* for 20 min at 4 °C and the pellet was removed. The mixture was again centrifuged at 12 000 × *g* for 30 min at 4 °C, the supernatant was removed, and the pellet was resuspended in TBS (pH 6.0) containing 0.1% (w/v) poly (ethylene glycol) 20000 and 0.01% (w/v) NaN_3_. The absorption peaks of the colloidal gold particles and gold–rSPG conjugate were detected using a microplate reader (Tecan, Mannedorf, Switzerland).

### Preparation of the GICA strips

The gold–rSPG conjugate was applied onto glass fiber membranes (9 mm in width) at a volume of 60 μl/cm and dried in vacuum using a freeze dryer (Thermo, Waltham, MA, USA). Then, by using an XYZ Biostrip Dispenser (Bio-Dot, Irvine, CA, USA), 0.5 mg/ml SEA of *S. japonicum* [10] and 0.5 mg/ml rSPG were transferred onto the NC membrane at a volume of 1 μl/cm to form the test and control lines, respectively. Subsequently, the membrane was dried in a biochemical incubator (Shanghai Boxun Medical Biological Instrument Corp, China) for 2 h at 37 °C. The coated membrane, conjugate pad, sample pad, and absorbent pad were laminated and pasted onto a plastic-backed support card with a 1–2 mm overlap of each component. The entire assembled scale board was cut length-wise and divided into strips measuring 3 × 60 mm using a guillotine cutter (CM4000 Guillotine, Bio-Dot). Finally, the strips were placed in a plastic card box, which in turn was put into an aluminum foil bag containing silica gel desiccant, and stored at room temperature.

### Lower limit of detection of the GICA strips

The lower limit of detection of the GICA strip was determined using serially diluted reference *S. japonicum*-positive serum (diluted with 0.9% NaCl (pH 7.2) at ratios from 1∶5 to 1∶1 280). The *S. japonicum*-negative buffalo serum was used as the negative control and 0.9% NaCl (pH 7.2) was employed as the blank control. The procedure was repeated more than three times. The 10 *S. japonicum*-positive buffalo serum samples were mixed and used as positive serum, and the 10 *S. japonicum*-negative buffalo serum samples were mixed and used as negative serum.

### Comparison of the sensitivity, specificity, and cross-reaction of the GICA strips with those of ELISA

The sensitivity and specificity of the GICA strips were verified using positive serum samples from 50 mice, 30 rabbits, and 18 buffaloes and negative serum samples from 20 mice and 20 rabbits. The serum samples from mice and rabbits were considered as positive if worms perfused from the portal vein. The clinical evaluations of the GICA strips were performed using serum samples from 73 goats and 80 buffaloes that presented hatching *S. japonicum* miracidia in their stool and from 44 goats and 52 buffaloes without hatching *S. japonicum* miracidia. The serum samples that were positive for various pathogens other than *S. japonicum*, including 37 *Orientobilharzia*-positive goat sera, 12 *H. contortus*-positive goat sera, and 14 *Paramphistomum*-positive buffalo sera, were used to evaluate the cross-reaction of the GICA strips. A total of 5 μl of the serum sample were mixed with 95 μl of 0.9% NaCl (pH 7.2) and stored at −20 °C until analysis. As the blank control, 0.9% NaCl (pH 7.2) was employed. Each sample was tested in triplicate using the GICA strip.

Meanwhile, the samples were also examined using ELISA for comparing the sensitivity, specificity, and cross-reaction of the GICA strips. Based on the checkerboard titration analysis, the wells of the microtiter plates (Costar, Acton, MA, USA) were coated with 15 μg/ml SEA diluted with carbonate-bicarbonate buffer (pH 9.6) and incubated overnight at 4 °C. Then, the wells were blocked with 1% (w/v) gelatin/PBST for 2 h at 37 °C and washed thrice with PBST for 5 min. Subsequently, mice, rabbit, buffalo, or goat serum samples at a dilution of 1∶100 with PBST were added to the wells (100 μl/well) and incubated for 2 h at 37 °C, and then washed thrice for 5 min with PBST. Each serum sample was added to three wells in one test. The HRP-conjugated goat anti-mouse IgG diluted at a ratio of 1∶2 500 with PBST, HRP-conjugated goat anti-rabbit IgG diluted at a ratio of 1∶2 500 with PBST, HRP-conjugated rabbit anti-goat IgG diluted at a ratio of 1∶4 000 with PBST, and HRP-conjugated goat anti-bovine IgG diluted at a ratio of 1∶4 000 with PBST were individually added to wells (100 μl/well) and the plates were incubated at 37 °C for 1 h. After that, the plates were washed thrice for 10 min with PBST, 3,3′,5,5′-tetramethylbenzidine dihydrochloride was added to each well (100 μl/well), and the reaction was stopped after 10 min using 2 M sulfuric acid (50 μl/well). The OD at 450 nm was determined using a microplate reader (Tecan, Mannedorf, Switzerland). All the tests were performed with reference negative and positive serum controls. The ELISA results were considered positive when the reading of the serum sample was 2.1 times higher than that of the negative control at an OD of 450 nm.

### Stability of the GICA strips

To establish the stability of the GICA strips, several of the conjugated reagents were stored for 3, 6, 9, and 12 months at room temperature. The stored strips were re-examined for specificity and sensitivity with known *S. japonicum*-positive and *S. japonicum*-negative buffalo sera.

### Statistical analysis

The *K*_*a*_ is a parameter that measures the strength of interactions between the molecules, the higher the *K*_*a*_, the greater the strength of interactions between the molecules. The same protein may have different *K*_*a*_ values with a variety of antibodies. The 2.1 times of the mean absorbance value of the reference negative sera was set as the cutoff value. A sample was considered positive when its mean absorbance value was higher than the cutoff value. The sensitivity, specificity, and cross-reactivity of GICA were compared with those of ELISA using the chi-square test. Sensitivity and specificity data were calculated as follows: sensitivity = number of true positives/(number of true positives + number of false negatives) and specificity = number of true negatives/(number of true negatives + number of false positives). Confidence intervals (*CI*) of 95% were applied to the data on sensitivity, specificity, and cross-reactivity. Stata software (version 13/SE) was used to perform the analysis. *P*-values < 0.05 were considered statistically significant.

## Results

### Expression, purification and identification of rSPG

**Fig. 2:**
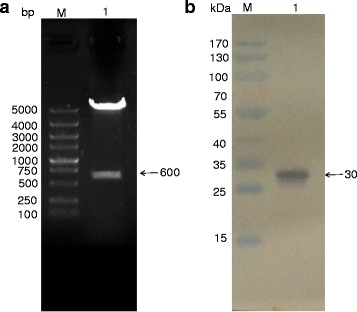
**a** Double digestion of the pET-28a(+)-rSPG. M: Marker DL5000 DNA ladder; Lane 1: Recombinant plasmid digested with restriction enzymes. **b** Western blot analysis of the rSPG; M: Protein marker; Lane 1: Purified rSPG recognized with HRP-conjugated goat anti-rabbit IgG

To obtain colloidal gold–protein, the 600-bp SPG were obtained, sequenced and cloned into expression vectors (Fig. 2a). Subsequently, western blot analysis was employed to identify the rSPG by using HRP-conjugated goat anti-rabbit IgG (Fig. 2b).

### Affinity constant of rSPG with the IgG from different animals

**Fig. 3:**
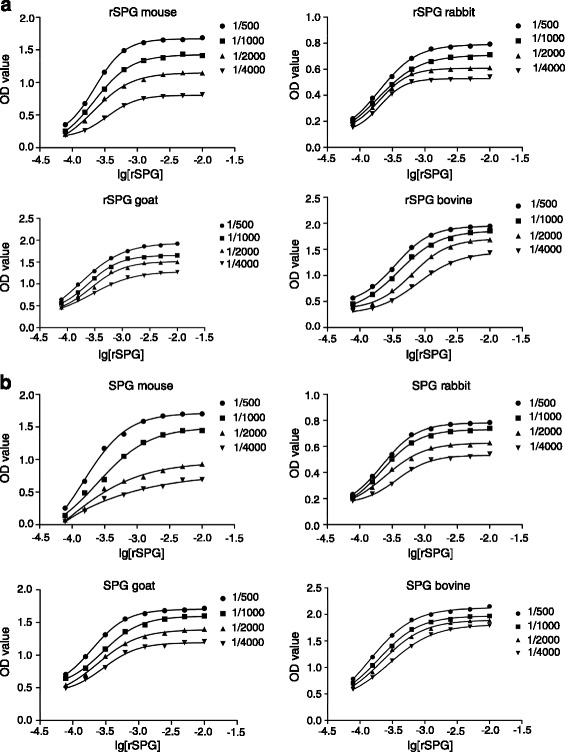
Determination of *K*_a_ of (**a**) rSPG and (**b**) SPG with the IgG from different animals

The *K*_*a*_ of rSPG with the IgG from different animals was determined by ELISA (Fig. 3). The *K*_a_ was calculated and the average *K*_a_ values of rSPG and SPG are shown in Table 1. No significant difference was noted between the *K*_a_ of rSPG and SPG (*P* > 0.05).

**Table 1:** *K*_a_ of rSPG and SPG with the IgG from different animals

	rSPG	SPG
Bovine	3.95 × 10^7^	1.07 × 10^8^
Goat	1.05 × 10^8^	1.24 × 10^8^
Rabbit	1.48 × 10^8^	8.84 × 10^7^
Mouse	9.01 × 10^7^	3.72 × 10^7^

### Measurement of the size of colloidal gold–rSPG particles

**Fig. 4:**
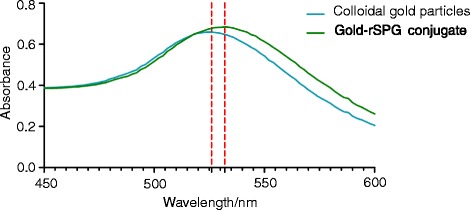
The absorption peaks of the colloidal gold particles and gold–rSPG conjugate

In the present study, gold particles were synthesized via chemical condensation, and the visible spectrum of the colloidal gold particles showed a maximum absorbance at 526 nm. Furthermore, maximum absorbance of the gold–rSPG conjugate was noted at a wavelength of 532 nm (Fig. 4).

### Schematic illustration of the GICA strips

**Fig. 5:**
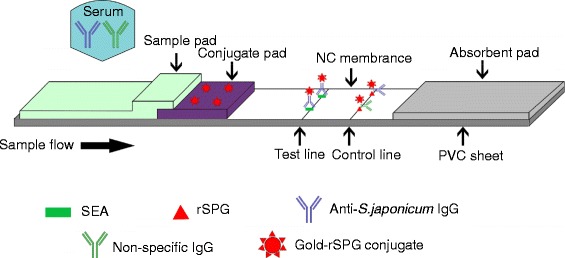
Schematic illustration of GICA. The serum is loaded onto the sample pad and the gold–rSPG conjugate is added onto the conjugate pad. The schistosome SEA is immobilized as the test line in the NC membrane. The rSPG is used as the control line. Following the application of a serum sample containing specific anti-*S. japonicum* IgG and non-specific IgG onto the NC membrane, the conjugated anti-*S. japonicum* IgG complex is captured by the SEA on the test line (T), resulting in a red band. The conjugated anti-*S. japonicum* IgG and nonspecific IgG are captured by the rSPG on the control line (C), resulting in a red band

The principle of the GICA is illustrated in Fig. 5. The positive result was indicated by the appearance of two red bands in the test (marked “T”) and control (marked “C”) lines. The negative result was represented by the appearance of only a single red band in the control line. The test was considered invalid if no red band was found or only one red band appeared in the test line.

### Lower limit of detection of the GICA strips

**Fig. 6:**
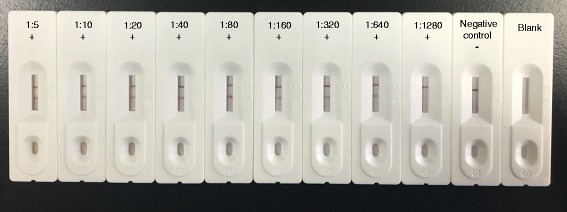
Lower limit of detection of the GICA strips. The *S. japonicum*-positive buffalo serum samples were serially diluted with 0.9% NaCl (pH 7.2) at ratios from 1:5 to 1:1280 and tested using GICA strips to determine the assay’s sensitivity. *S. japonicum*-negative buffalo serum sample was used as the negative control and 0.9% NaCl (pH 7.2) was used as the blank. Three independent experiments were performed in triplicate

The lower limit of detection of the GICA strip was shown in Fig. 6, the red band can be clearly observed on the test line of 1∶640 dilution. However, when the dilution was 1∶1 280 or lower, only one red band can be noted on the negative control. This finding indicated that the GICA strip could detect low titer of antibodies in the serum samples. Similar results were observed upon repeating the tests more than three times, thus revealing the high reproducibility of the results obtained using the GICA strip.

### Cross-reaction of the GICA strips

The cross-reaction of the GICA strips was shown in Table 2, the cross-reaction of the GICA strips with *Paramphistomum* sp. in buffaloes was 14.29% (2/14, 95% *CI*: 1.78%–42.81%), which was lower than that of ELISA (50.00%, 7/14, 95% *CI*: 23.30%–76.96%), whereas both GICA and ELISA presented a cross-reaction of 16.67% with *H. contortus* in goats (2/12, 95% *CI*: 2.09%–48.41%)*.* However, the cross-reaction of the GICA strips with *Orientobilharzia* sp. in goats was 33.33% (12/36, 95% *CI*: 18.56%–50.97%), which was significantly lower than that of ELISA (88.89%, 32/36, 95% *CI*: 73.94%–96.89%) (χ^2^ = 23.377, *P* < 0.01).

**Table 2:** Cross-reaction of the GICA and ELISA

Serum	No. of cases	GICA		ELISA	
		No. of positive	Positive rate (%)	No. of positive	Positive rate (%)
Serum from *Paramphistomia*-infected buffalo	14	2	14.29	7	50.00
Serum from *H. contortus*-infected goat	12	2	16.67	2	16.67
Serum from *Orientobilharzia*-infected goat	36	12	33.33^*^	32	88.89

^*^: *P* < 0.01, for the GICA strip, when compared with ELISA

### Sensitivity and specificity of the GICA strips

The sensitivity of both the GICA strips and ELISA was 100% for 50 mice (50/50, 95% *CI*: 92.89%–100.00%) and 30 rabbit (30/30, 95% *CI*: 88.43%–100.00%) serum samples with *S. japonicum* infection, and the specificity of both the GICA strips and ELISA was 100% for 20 mice and 20 rabbit (20/20, 95% *CI*: 83.16%–100.00%) serum samples without *S. japonicum* infection (Table 3).

**Table 3:** Results of diagnosis of schistosomiasis in mice, rabbits, buffaloes and goats using GICA and ELISA

Serum	No. of cases	GICA		ELISA	
		No. of positive	Positive rate (%)	No.of positive	Positive rate (%)
Serum from *S. japonicum*-infected mice	50	50	100.00	50	100.00
Serum from uninfected mice	20	0	0.00	0	0.00
Serum from *S. japonicum*-infected rabbit	30	30	100.00	30	100.00
Serum from uninfected rabbit	20	0	0.00	0	0.00
Serum from *S. japonicum*-infected buffalo	80	80	100.00	80	100.00
Serum from uninfected buffalo	52	3	5.77	8	15.38
Serum from *S. japonicum*-infected goat	73	73	100.00	73	100.00
Serum from uninfected goat	44	5	11.36	11	25.00

**Fig. 7:**
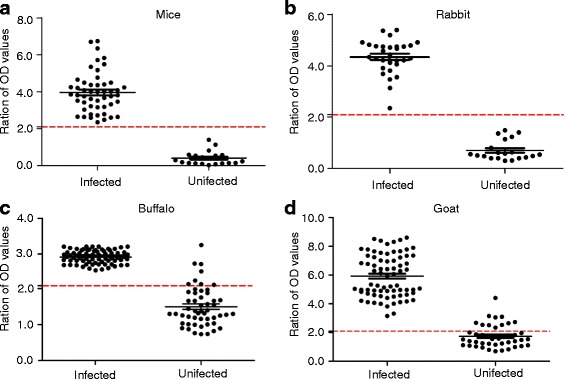
**a** Schistosomiasis diagnosis in **a** mice, **b** rabbits, **c** buffaloes and **d** goats using ELISA

Furthermore, buffalo and goat serum samples were employed to compare the sensitivity and specificity of GICA with those of ELISA in detecting *S. japonicum.* The serum samples from buffaloes and goats were determined as positive by fecal miracidium hatching test. The sensitivity of both the GICA strip and ELISA was 100% (80/80, 95% *CI*: 95.49%–100.00%) for the positive samples from buffaloes, whereas the specificity of the GICA strip was higher (94.23%, 49/52, 95% *CI*: 84.05%–98.79%) for the samples from uninfected buffaloes, when compared with that of ELISA (84.62%, 44/52, 95% *CI*: 71.92%–93.12%). Nevertheless, there was no significant difference between GICA and ELISA with regard to schistosomiasis diagnosis using buffalo serum (*χ* ^2^ = 0.148, *P* > 0.05). Similarly, the sensitivity of both GICA and ELISA was 100% (73/73, 95% *CI*: 95.07%–100.00%) for the positive goat serum samples, whereas the specificity of GICA was higher (88.64%, 39/44, 95% *CI*: 75.44%–96.21%) for the samples from uninfected goats, when compared with that of ELISA (75.0%, 33/44, 95% *CI*: 59.66%–86.81%) (Table 3 and Fig. 7). However, no significant difference was noted between GICA and ELISA with respect to schistosomiasis diagnosis using goat serum (*χ* ^2^ = 0.415, *P* > 0.05).

**Fig. 8:**
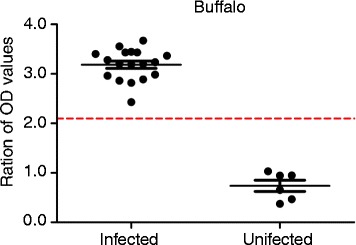
ELISA results for *S. japonicum* detection in the serum samples from buffaloes with various infection intensities

To assess the potential of GICA and ELISA as a diagnostic tool for detecting schistosomiasis in buffaloes, serum samples from 18 *S. japonicum*-infected buffaloes with various infection intensities and six uninfected buffaloes were employed. When the infection was less than 20 worms per buffalo, the sensitivity of GICA was lower (75%), when compared with that of ELISA (100%). However, when the infection was more than 20 worms per buffalo, the sensitivity of both GICA and ELISA was 100% (Table 4 and Fig. 8). Nevertheless, there was no significant difference between GICA and ELISA in the diagnosis of schistosomiasis in buffaloes with various infection intensities (*χ* ^*2*^ = 0.76, *P* > 0.05).

**Table 4:** Detection of *S. japonicum* in the serum samples from buffaloes with various infection intensities using GICA and ELISA

No. of parasites/buffalo	No. of cases	GICA		ELISA	
		No. of positive	Positive rate (%)	No. of positive	Positive rate (%)
0	6	0	0.00	0	0.00
<20	4	3	75.00	4	100.00
20–50	6	6	100.00	6	100.00
50–80	5	5	100.00	5	100.00
>80	3	3	100.00	3	100.00

### Stability of the GICA strips

The results showed that GICA strips stored at room temperature for 12 months and retained their sensitivity and required only 5 μl of the positive serum samples from buffaloes or goats, similar to freshly produced GICA strips.

## Discussion

*S. japonicum* has a wide range of mammalian hosts, including humans, buffaloes, cattle, goats, sheep and pigs, etc., which further complicates the control of schistosomiasis [11]. By the end of 2012, it had been estimated that around 1.03 million cattle and buffaloes, 2.02 million goats and sheep, and 0.89 million other domestic animals were infected with *S. japonicum* in schistosomiasis-endemic regions in China [12]. Schistosomiasis in domestic animals not only causes heavy economic losses, but the stool of the infected animal acts as the most important source of infection. Thus, unless schistosomiasis epidemic is effectively controlled in domestic animals, complete control and elimination may not be feasible [13]. Furthermore, the quarantine and monitoring of the disease is not easy and requires long periods of time.

The GICA is most widely used for the detection of a variety of diseases [14, 15]. The application of colloidal gold in immunology is based on the principle of antigen–antibody reaction. In this study, colloidal gold was conjugated with rSPG. SPG is a streptococcal cell wall protein with the capability of binding to a variety of human and animal IgG antibodies. It was first reported in 1973 by Kronvall. Later, in 1984, Bjorck named, separated, and purified SPG [16]. Then, SPG–gold was combined with several monoclonal or polyclonal antibodies for locating a variety of antigenic sites [9]. The IgG-binding molecule, SPG, is prepared from the cell of a group G streptococcal strain. Some regions of homology have been reported in the structure of SPG. The C domain (containing C1, C2, and C3 domains) of SPG in the COOH-terminus has been noted to have affected the binding of SPG to IgG [17]. While the C1 and C2 domains differ in only two amino acids, the C1 and C3 domains have six amino acid inconsistencies. The IgG-binding capacity of the C3 domain has been found to be seven times higher than that of the C1 domain [18]. Therefore, in the present study, rSPG containing only the C3 domain was developed. Besides, western blot analysis was employed to identify the ability of rSPG to bind to IgG.

In this study, there was no significant difference between the *K*_a_ of rSPG and SPG, although the rSPG contained only the amino acids of the C3 domain of SPG, which can specifically bind to the Fc fragments of IgG. Besides, it must be noted that the amino acids of the A and B domains of SPG can specifically bind to the Fab fragments of IgG and human serum albumin, affecting the normal binding of antibody to antigen. Moreover, the binding of SPG to human serum albumin may lead to a false-positive result. Thus, the rSPG was developed to overcome these drawbacks and reduce non-specific binding or cross-reaction.

In the present study, the maximum absorbance of the colloidal gold particles was noted at a wavelength of 526 nm and the size of the colloidal gold particles was about 25 nm [10]. As described in previous reports [19], the optimal particle size of colloidal gold for most of the diagnostic applications is 20–40 nm because of the tradeoff between required visibility and steric hindrance. GICA strips were used to detect schistosomiasis based on an indirect immunoassay format. It must be noted that the specific anti-*S. japonicum* IgG in the positive serum samples reacted with the gold–rSPG conjugate to form a gold–rSPG–anti-*S. japonicum* antibody complex, which was captured by SEA on the NC membrane to form a gold–rSPG–anti-*S. japonicum* antibody–SEA complex that generated a red band on the test line. The density of the red band was proportional to the concentration of the anti-*S. japonicum* antibodies. Excess gold–rSPG conjugate reacted with the nonspecific IgG or specific anti-*S. japonicum* antibodies in the serum sample, flowed over the test line, and bound to the purified rSPG on the control line (marked “C”), forming another red band on the control line of the strip.

The GICA strip could detect schistosomiasis in four animal species (mice, rabbit, goat, and buffalo) and requires only a small volume of serum (5 μl) for detection. The results can be assessed by the naked eye. It must be noted that most of the other rapid diagnostic strips can be applied only to human serum samples [20, 21], require special equipment [22], can be applied only to one animal species, or require larger volumes of serum samples (50 μl) [23].

The cross-reaction of GICA with *Paramphistomum* sp. and *Orientobilharzia* sp. (14.29% and 33.33%, respectively) was lower than that of ELISA (50.00% and 88.89%, respectively). Furthermore, the cross-reaction of both GICA and ELISA with *H. contortus* was 16.67%*.* The sensitivity of both GICA and ELISA was 100% for mice, rabbit, buffalo, and goat serum samples. However, while the specificity of both GICA and ELISA was 100% for mice and rabbit serum samples, the specificity of GICA was higher for samples from uninfected buffaloes and goats (94.23% and 88.64%, respectively), when compared with that of ELISA (84.62% and 75.0%, respectively). Besides, it can be concluded that the GICA and ELISA did not present any significant differences in the detection of schistosomiasis in animals using serum samples (*P* > 0.05). The high degree of consistency observed between the GICA and ELISA supports the reliability of the novel test strip.

It must be noted that in the present study, the serum samples of mice and rabbits were obtained and artificially infected with *S. japonicum* in our laboratory, whereas the serum samples from *S. japonicum*-positive buffaloes and goats were obtained from schistosomiasis-endemic areas and those from *S. japonicum*-negative buffaloes and goats were obtained from schistosomiasis-non-endemic areas. Among the negative goat and buffalo serum samples, five samples from goats and three samples from buffaloes were found to show positive results, which may be owing to some cross-reaction. It must be noted that the SEA of *S. japonicum* can cross-react with the antibodies to other parasitic flukes or soil-transmitted helminths, thereby substantially lowering the specificity of the GICA strips [24, 25].

The stability of the GICA strips suggested that the validity period of the GICA strips, with no loss of sensitivity and specificity with respect to detection of *S. japonicum*, was at least 12 months at room temperature. Besides, as no negative sample showed a false-positive result, it can be concluded that the specificity of the GICA strips with regard to *S. japonicum* detection did not change.

## Conclusion

A novel GICA strip was successfully developed and preliminarily applied for the detection of schistosomiasis in various domestic animals. The strip requires only 5 μl of serum sample for detection and the results can be assessed within 5 min with the naked eye. Furthermore, when compared with the conventional ELISA method, the GICA strip presented higher sensitivity and specificity. Moreover, unlike the intuitive assessment of the results obtained using conventional ELISA and IHA methods, diagnosis using GICA is simple and does not require special equipment. Thus, the GICA may be a useful tool for large-scale screening of schistosomiasis in domestic animals in endemic areas.

## Additional file

Additional file 1: Multilingual abstract in the six official working languages of the United Nations. (PDF 414 kb)

## Abbreviations

ELISA: Enzyme linked immunosorbent assay
GICA: Colloidal gold immunochromatography assay
*Ka*: Affinity constant
rSPG: Recombinant streptococcal protein G
SEA: Schistosome soluble egg antigen
SPA: Staphylococcal protein A
SPG: Streptococcal protein G
TEM: Transmission electron microscopy
