# Supplementary material for: A novel colloidal gold immunochromatography assay strip for the diagnosis of schistosomiasis japonica in domestic animals
Source: Infect Dis Poverty. 2017 Apr 7;6:84. doi: 10.1186/s40249-017-0297-z (PMC5384140; doi:10.1186/s40249-017-0297-z)
Supplement: Additional file 1: — Multilingual abstract in the six official working languages of the United Nations. (PDF 414 kb) [file 40249_2017_297_MOESM1_ESM.pdf]

Translation of the abstract into the six official working languages of the United Nations

فحص شريط استشراب مناعي ذهب غرواني جديد لتشخيص مرض البلهارسيا اليابانية في الحيوانات الداجنة

روي شو، جينتاو فينغ، هونغ يانغ، تشاو لوفي،دينجوين تشاو، جياوجياو لين، كه لو، هاو لي، جينمينج ليو، شياودان تساو، تاو وانغ، جينلي تساي، تشاوجي وانغ، بينجوانج جيا، شيان هان، تشونجانج تشو

الملخص

الخلفية: تظل البلهارسيا مصدر قلق صحي عام رئيسي في الصين وقد كشف المسح الوبائي عن أن البقريات والماعز المصابة هي المصادر الرئيسية لإنتقال المرض ولذلك فإنه من الضروري تطوير تقنية حساسة لتشخيص مرض البلهارسيا في الحيوانات الداجنة.

الأساليب: تم تطوير فحص شريط استشراب مناعي ذهب غرواني (GICA) جديد للكشف عن البلهارسيا اليابانية في الحيوانات الداجنة حيث تم دمج الذهب الغرواني مع البروتين العقدي المصنع ج (rSPG) وللمقارنة بين المجموعة محل الإختبار والمجموعة الضابطة تم مسح مستضد بيض البلهارسيا المذاب والبروتين العقدي المصنع ج علي غشاء نتروسلولوزي.

النتائج: كان أقل تخفيف من مصل الدم المكتشف من الجاموس المصابة هو 1:640 وكان معدل التفاعل المتداخل لشريط الاستشراب المناعي الذهب غرواني مع الديدان المثقوبة من نوع البارامفستومم هو 14.29٪ في الجاموس و16.67٪ مع الديدان المدورة من نوع هيمونكس في الماعز و 33.33٪ مع الديدان من نوع الأورينتوبلهارسيا في الماعز وتعتبر هذه النتائج أقل قليلاً أو مماثلة لتلك التي تم الحصول عليها عن طريق إختبار قياس الممتز المناعي المرتبط بالإنزيم (إليزا) وعلاوة على ذلك أظهرت الشرائط المستخدمة للكشف عن البلهارسيا اليابانية في الفئران والأرانب والجاموس والماعز حساسية عالية بمعدلات 100٪ و 100٪ و 94.23٪ و 88.64٪ علي التتابع كما أن حساسية أو خصوصية شرائط الاستشراب المناعي الذهب غرواني لم تظهر إختلافات كبيرة بعد تخزينها لمدة عام في درجة حرارة الغرفة و بالمقارنة مع الأليزا أظهرت الشرائط حساسية ودقة مماثلة في تشخيص مرض البلهارسيا في الفئران والأرانب والجاموس والماعز وبالإضافة إلي ذلك فإن الإختبار يحتاج فقط إلي 5 ميكروليتر من مصل الدم والكشف يمكن أن يتم إنجازه في غضون 5 دقائق.

الخلاصة: هذه الدراسة هي الأولى من نوعها لتطوير شريط استشراب مناعي ذهب غرواني مقترن ببروتين عقدي مصنع لتشخيص مرض البلهارسيا في الحيوانات الداجنة وأظهرت النتائج الأولية ان الشريط المطور ربما يكون مناسب في إجراء فحص على نطاق واسع لمرض البلهارسيا في المناطق الموبوءة..

Translated from English version into Arabic by Mohamed R. Habib

一种新型的用于诊断家畜日本血吸虫病的胶体金免疫层析试纸条

许瑞，冯金涛，洪炀，吕超，赵登云，林娇娇，陆珂，李浩，刘金明，曹晓丹，王涛，宰金丽，王钊哲，贾秉光，韩倩，朱传刚。

摘要

引言：在中国，血吸虫病仍然是一个十分重要的公共卫生问题，流行病学调查显示患病牛羊是血吸虫病最重要的传染源。因此，有必要研制可以同时检测多种动物血吸虫病的快速敏感的试剂盒。

方法：本实验研制的胶体金免疫层析试纸条可以检测家畜日本血吸虫病。此试纸条以胶体金颗粒标记重组蛋白 G（rSPG）来捕获抗体，以虫卵可溶性抗原（SEA）和 rSPG 分别作为检测线和质控线。

结果：此试纸条的最低检测限为 1:640 倍稀释的阳性水牛血清。其与前后盘吸虫交叉反应率为 14.29%；与捻转血矛线虫交叉反应率为 16.67%；与东毕吸虫的交叉反应率为 33.33%，均低于 ELISA 的检测结果。此外，该试纸条在室温放置 12 个月后其检测结果的敏感性和特异性均没有

发生变化。该试纸条检测日本血吸虫感染的小鼠、兔、水牛和山羊血清时的显示出了高度的敏感性和特异性。其中，敏感性均为 100.00%，特异性分别为 100.00%，100.00%，94.23%和 88.64%。并且，GICA 法与 ELISA 法在检测这四种动物的血清时所得到的结果无统计学差异。最后，该方法仅用 5 μl 血清即可在 5 分钟内得到检测结果，是一种简便快速的检测方法。
结论：本研究首次将重组蛋白 G 与胶体金颗粒相结合，研制出了能够成功诊断家畜日本血吸虫病的胶体金免疫层析试纸条（GICA）。研究显示，此试纸条适合疫区血吸虫病的大规模筛查时应用。

Translated from English version into Chinese by Rui Xu

Nouveau test bandelette immunochromatographique à l’or colloïdal pour le diagnostic de la schistosomiase japonicum chez les animaux domestiques

Rui Xu, Jintao Feng, Yang Hong, Chao Lv, Dengyun Zhao, Jiaojiao Lin, Ke Lu, Hao Li, Jinming Liu, Xiaodan Cao, Tao Wang, Jinli Zai, Zhaozhe Wang, Bingguang Jia, Qian Han, Chuangang Zhu

Résumé

Contexte: La schistosomiase demeure un problème majeur de santé publique en Chine, et une enquête épidémiologique a révélé que les bovins et les chèvres infectés par le schistosome sont les principales sources de transmission de la maladie. Par conséquent, l’élaboration de techniques sensibles pour le diagnostic de la schistosomiase chez les animaux domestiques est nécessaire.

Méthodes: Un nouveau test bandelette immunochromatographique à l’or colloïdal (GICA) a été développée pour détecter *Schistosoma japonicum* chez les animaux domestiques. L'or colloïdal a été conjugué à une protéine streptococcique recombinante G (rSPG). Comme le test et les bandes de contrôle, l'antigène soluble des œufs de schistosome et la proteine rSPG, respectivement, ont été transférés sur une membrane de nitrocellulose.

Résultats: La dilution de sérum la plus faible détectée était de 1:640 pour les buffles infectés par la schistosomiase. Le taux de réaction croisée du GICA était de 14,29 % avec *Paramphistomum sp*. chez les buffles, 16,67 % avec *Haemonchus sp*. chez les chèvres, et 33,33 % avec *Orientobilharzia sp*. chez les chèvres. Ces résultats étaient légèrement inférieurs et semblables à ceux obtenus par ELISA. De plus, les bandelettes pour la détection de *S. japonicum* chez les souris, les lapins, les buffles et les chèvres ont présenté une sensibilité élevée (100,00 %, 100,00 %, 100,00 % et 100,00 %, respectivement) et une spécificité de 100,00 %, 100,00 %, 94,23 % et 88,64 %, respectivement. En plus, la sensibilité et la spécificité des bandelettes GICA n'a pas présenté de différences significatives après 12 mois de stockage à température ambiante. Comparativement à l'ELISA, les bandelettes GICA présentaient une sensibilité et une spécificité similaires dans le diagnostic de la schistosomiase chez la souris, le lapin, le buffle et la chèvre. En outre, seulement 5 μl de sérum sont nécessaires pour le test et la détection peut être achevée en 5 minutes.

Conclusion: Cette étude est la première à développer un test bandelette immunochromatographique utilisant un conjugué or-rSPG pour le diagnostic de la schistosomiase chez les animaux domestiques. Les résultats préliminaires montrent que la bandelette développée peut convenir au dépistage à grande échelle de la schistosomiase dans les zones endémiques.

Translated from English version into French by GARBA DJIRMAY Amadou

Новейшие иммунохроматографические экспресс-тесты с коллоидным золотом для диагностики японского шистосомоза у домашних животных

Rui Xu, Jintao Feng, Yang Hong, Chao Lv, Dengyun Zhao, Jiaojiao Lin, Ke Lu, Hao Li, Jinming Liu, Xiaodan Cao, Tao Wang, Jinli Zai, Zhaozhe Wang, Bingguang Jia, Qian Han, Chuangang Zhu

Аннотация

Справочная информация: Шистосомоз остается серьезной проблемой общественного здравоохранения в Китае. эпидемиологическое исследование показало, что быки и козы, инфецированные шистосомозом, являются основным источником передачи заболевания. Таким образом, стала очевидной необходимость разработки чувствительного метода диагностики шистосомоза у домашних животных.

Методология: Новейший иммунохроматографический экспресс-тест с коллоидным золотом (GICA) был разработан для обнаружения *Schistosoma japonicum* у домашних животных. Коллоидное золото связывалось с рекомбинантныйм белком стрептококка группы G (rSPG). Антиген растворенного яйца шистосомы и rSPG, соответственно, были перенесены на нитроцеллюлозную мембрану.

Результаты: Минимально отмеченный уровень разведения сыворотки крови составил 1:640 для буйволов, зараженных шистосомозом. Уровень перекрестных реакций при тесте GICA составил 14.29% с *Paramphistomum* sp. у буйволов, 16.67% с *Haemonchus* sp. у коз и 33,33% с *Orientobilharzia* sp. также у коз. Эти результаты были незначительно ниже или похожи на те, что были получены с помощью теста ELISA. Кроме того, полоски для обнаружения *S. japonicum* у мышей, кроликов, буйволов и коз показали высокую чувствительность (100.00%, 100.00%, 100.00% и 100,00%, соответственно) и специфичность (100.00%, 100.00%, 94.23% и 88.64%, соответственно). Также чувствительность и специфичность полосок теста GICA не продемонстрировала каких-либо существенных различий после хранения в течение 12 месяцев при комнатной температуре. По сравнению с ELISA, полоски GICA показали сходную чувствительность и специфичность при диагностике шистосомоза у мышей, кроликов, буйволов и коз. Кроме того, всего 5 мкл сыворотки необходимы для тестирования и обнаружения. Тест может быть выполнен в течение 5 минут.

Вывод: Данное исследование является первым исследованием по диагностике шистосомоза у домашних животных, ставящим перед собой цель разработать полоски-тесты GICA с использованием связи между коллоидным золотом и rSPG. Предварительные результаты показали, что разработанные полоски-тесты могут быть пригодны для широкомасштабного скрининга шистосомоза в эндемичных районах.

Translated from English version into Russian by Dmitry Esin

Una nueva tira reactiva de inmunocromatografía de oro coloidal para el diagnóstico de schistosomiasis japonica en animales domésticos

Rui Xu, Jintao Feng, Yang Hong, Chao Lv, Dengyun Zhao, Jiaojiao Lin, Ke Lu, Hao Li, Jinming Liu, Xiaodan Cao, Tao Wang, Jinli Zai, Zhaozhe Wang, Bingguang Jia, Qian Han, Chuangang Zhu

RESUMEN

Antecedentes: La schistosomiasis sigue siendo una preocupación importante en la salud pública en China y un estudio epidemiológico ha revelado que los bovinos y las cabras infectados con schistosomas son las principales fuentes de transmisión de la enfermedad. Por consiguiente, es necesario el desarrollo de una técnica sensible para el diagnóstico de la schistosomiasis en animales domésticos.

Método: Se desarrolló una nueva tira reactiva de inmunocromatografía de oro coloidal (GICA) para detectar *Schistosoma japonicum* en animales domésticos. El oro coloidal se conjugó con la proteína recombinante G estreptocócica (rSPG). Como líneas de ensayo y de control, el antígeno soluble del huevo de *Schistosoma* y rSPG, respectivamente, se transfirieron a membrana de nitrocelulosa.

Resultados: La dilución de suero detectable más baja fue 1:640 para búfalos infectados con schistosomas. La tasa de reacción cruzada de GICA fue del 14,29% con *Paramphistomum* sp. en búfalos, del 16,67% con *Haemonchus* sp. en cabras, y del 33,33% con *Orientobilharzia* sp. en cabras. Estos resultados fueron ligeramente inferiores y similares a los obtenidos mediante ELISA. Además, las tiras reactivas para detectar *S. japonicum* en ratones, conejos, búfalos y cabras mostraron una alta sensibilidad (100,00%, 100,00%, 100,00% y 100,00%, respectivamente) y especificidad (100,00%, 100,00%, 94,23% y 88,64%, respectivamente). Y la sensibilidad o especificidad de las tiras reactivas GICA no presentaron diferencias significativas después de su almacenamiento durante 12 meses a temperatura ambiente. En comparación con ELISA, las tiras reactivas GICA mostraron sensibilidad y especificidad similares en el diagnóstico de schistosomiasis en ratones, conejos, búfalos y cabras. Además, sólo se requieren 5 µl de suero para la prueba y la detección puede completarse en 5 minutos.

Conclusión: Este estudio es el primero en desarrollar una tira reactiva GICA que usa un conjugado oro-rSPG para el diagnóstico de la schistosomiasis en animales domésticos, y los resultados preliminares mostraron que la tira reactiva desarrollada puede ser idónea para el cribado a gran escala de la schistosomiasis en áreas endémicas.

Translated from English version into Spanish by Màrius V. Fuentes
